# (Re)Considering the jump scare in four elements

**DOI:** 10.3389/fpsyg.2025.1569394

**Published:** 2026-01-06

**Authors:** Elizabeth A. M. Acosta, David R. W. Sears

**Affiliations:** 1 Performing Arts Research Lab, Department of Interdisciplinary Arts, Texas Tech University, Lubbock, TX, United States; 2 Music Cognition & Computation Lab, Department of Music Theory, University of Michigan, Ann Arbor, MI, United States

**Keywords:** horror, film, cinema, expectation, startle response, jump scare

## 1 Introduction

References to jump scares and startle responses in the horror film literature routinely demonstrate a certain psychologizing impulse. According to Martin (2019), the jump scare that often provokes a startle reflex (SR) in audiences is perhaps “(t)he most well-used auditory (and visual) device in horror film(s)” (p. 5). Colloquially, the term *jump scare* refers to a sudden visual intrusion from offscreen combined with a sudden auditory outburst that ultimately intends to shock, surprise, or otherwise frighten the audience. Jump scares can serve as potentially ideal, ecologically rich stimuli for experimental studies exploring such topics as the defense cascade, the induction of emotions like fear and disgust, and selective exposure to negative stimuli (Bradley et al., 2001; Oliver, 2003). And yet, Baird (2000) laments that, “not a few philosophers and psychologists have been content to relegate (the) startle to the category of dumb reflex, little more dynamic than a sneeze or a knee jerk” (p. 13). This assumption can also be seen in recent psychological analyses of horror films that relegate the jump scare to simple cause-and-effect (e.g., Hye-Knudsen et al., 2024; Nummenmaa, 2024).

Perhaps for this reason, the experimental literature to date tends to focus on individual motivations for viewing the horror genre, rather than on addressing questions surrounding the perceptual and cognitive mechanisms underlying jump scares and the SRs they elicit. Why, for example, do jump scares stimulate intense emotional responses in spite of the audience's awareness of the art form's inherent fictionality? Similarly, if psychophysiological studies primarily associate the SR with fear, anger, and disgust (Bradley et al., 1999), why do horror films continue to fill theaters? Put more simply, why do we *like* scary movies?

To address these questions, we propose four elements of the jump scare that make it an ideal theoretical paradigm for experimental research in the years ahead.

## 2 A jump scare is an aesthetic experience that elicits a behavioral response

The startle response—characterized by blinking, accelerated heart rate, and pupil dilation—is broadly categorized as a “defensive reflex… potentiated during aversive states” (Bradley et al., 1999, p. 182). In experimental contexts, the startle response is reliably induced when a participant is exposed to negative imagery and/or unpleasant sounds, especially during a heightened fear state, as opposed to pleasant or neutral stimuli and their concomitant emotional states (Bradley and Lang, 2000; Lang et al., 1990; Vrana and Lang, 1990; Vrana et al., 1988). The startle response is often conceptualized as a proxy or measurable manifestation of fear and anxiety, where individuals in a higher state of physiological arousal produce stronger startle responses when presented with negatively valanced stimuli.

As Martin (2019) notes, the startle response lies at the heart of the jump scare, yet not all startles are jump scares. The concept of the jump scare originates and exists primarily within the context of the horror film genre. The first instance of the jump scare is often attributed to Val Lewton's *Cat People* (Lewton, 1942; Donnelly, 2005; Hutchings, 2009). At around the midpoint of the film, a woman is walking down the street alone at night when she hears a rustling in the bushes. Her heeled footsteps echo on the pavement as her pace quickens. The camera cuts between her face, her steps, and her obscured surroundings. She looks camera left, when suddenly a large object slams into view from camera right, accompanied by a shrill, cacophonous sound. Only after this initial shock does the woman (and by extension, the audience) realize that the object is a bus coming to an abrupt stop. Relieved, the woman climbs safely aboard.

This scene remained so impactful that for many years the phenomenon was referred to as the *bus effect*, and moments that were intended to mimic its effect on audiences— employed by both Lewton and others—were referred to as *busses* (Baird, 2000). Sometime later, the term was generalized to *jump scare*, and though the origin of the phrase is unknown, the filmic concept of the jump scare has remained surprisingly static. Hutchings (2009) likewise acknowledges that although there is certainly a commonality in terms of physiological response, jump scares, “are provoked within a very different context (than real-life shocks), one that is organized around suspense(,) and which is heavily dependent on audience expectations and competences” (p. 223). Just as SRs occur within the psychophysiological process of the *defense cascade* (Lang et al., 1997), jump scares typically occur within a particular type of film scene, termed the *threat scene* (Baird, 2000). Thus, in this case, a jump scare may elicit a specific variation of the SR, one that is elicited exclusively by an aesthetic experience. This distinction invites discussions of ecological validity, and whether the SR in the context of horror films operates differently than in everyday life, which, in turn, may differ from the SR elicited in traditional laboratory conditions.

Previous studies exploring the perception and cognition of music, for example, have suggested that the emotions induced by music (and other artforms) should be distinguished from “everyday emotions” elicited in nonmusical (or nonartistic) contexts (e.g., Konečni, 2008). From this point of view, basic emotions like happiness and fear are physiologically and functionally distinct, metabolically costly, and typically infrequent (~1–2 times per day; Ekman et al., 1985; Konečni, 2008). Conversely, the emotions induced by music have no clear referential cause, nor do they serve an obvious biological function (Scherer et al., 2002). For this reason, some studies have instead argued that music induces specific *aesthetic emotions*, such as aesthetic awe, being moved, or thrills/chills (Konečni, 2008; Scherer et al., 2002; c.f. Juslin and Västfjäll, 2008).

In the case of horror films, the initial intention and function of a jump scare is to frighten or disgust, and yet the audience is also aware, on some level, that the scare cannot physically harm or threaten them, which may instead lead to positive appraisals of initially negative physiological reactions (e.g., pleasure, enjoyment). From this point of view, then, how might the aesthetic environment of the horror film modulate or otherwise affect the SR and its subsequent appraisal? What aspects of jump scares have the strongest influence on the SR, and how might it differ from SRs elicited in “everyday,” nonaesthetic contexts, if at all?

Traditionally, the SR has been measured using a combination of heart rate, skin conductivity, and facial muscles associated with fear-potentiated startles such as the zygomatic and corrugator muscles (e.g., Lang et al., 1997). Therefore, studies exploring psychophysiological measures during jump scares and the SRs they elicit could prove valuable in assessing potential contextual differences with those elicited in nonaesthetic contexts. One study that could be easily adapted to the context of jump scares is Steinbeis et al.'s (2006) investigation of the effect of harmonic expectancy violations on emotion, which measured psychophysiological responses to Bach chorales. Examining the SR in response to multimodal, ecologically rich stimuli like jump scares using similar psychophysiological measures could provide insight into whether, and to what degree, the aesthetic environment of film affects the SR, while also revealing more about the psychological mechanisms involved in watching horror films.

## 3 A jump scare is multimodal

Jump scares implicate both visual and auditory information. Conventionally, jump scares include both sudden (often frightening and/or disgusting) imagery, combined with a discordant, loud acoustic *stinger*, both of which are central to the horror genre (Heimerdinger, 2012). Compared to the complex audiovisual experience of film, however, the interplay between the auditory and visual modalities in experimental studies of the startle response remains critically underexamined (Buhler, 2014). In multimodal experiments examining the SR, the selected stimuli often feature visual imagery tasks like imagining a frightening scenario (e.g., Angrilli et al., 1996; Bradley et al., 1999; Cook et al., 1991). The SR is then elicited by a probe tone of white noise. In Martin's (2019) review of 18 SR studies, for example, only three featured audiovisual stimuli (i.e. film scenes) (see Jansen and Frijda, 1994; Koukounas and McCabe, 2001; Kreibig et al., 2011). What is more, in each of these studies, the probe tone still elicited the startle, rather than the stimulus itself. Thus, to better understand the multimodal nature of jump scares and the SRs they elicit, the experimental literature might additionally draw inspiration from research in sound theory/design, film studies, and musicology.

Recent studies consider all sound elements in a film's “integrated soundtrack,” including not only dialogue/speech and music, but also sound effects, ambient noise, and even silence (Greene, 2016; Kulezic-Wilson, 2019; Segura, 2022). In the case of horror films, screeching violins or howling wind may contribute to the horror film's overall atmosphere of unease, while more dramatic, aggressive sound elements like a scream or a pounding drum can engender the acoustic stinger during moments of terror (Heimerdinger, 2012). This framework speaks to the complexity of acoustic information involved in jump scares, and although the current SR literature provides valuable insights concerning cross-modality, the white noise probe tones in the SR literature may not be ecologically comparable to the audiovisual context of jump scares (Lang et al., 1997).

This claim prompts a closer examination of the probe tone that elicits the SR. How does the simultaneous presentation of visual and auditory information affect the SR? Do the specific components of the probe alter the magnitude or overall organization of the SR in some way? Does the relatedness between the visual and auditory modalities in more ecologically rich stimuli affect this response (e.g., the noise of a chainsaw paired with a chainsaw-wielding killer, an inhuman scream paired with an open-mouthed ghostlike figure, etc.)?

## 4 A jump scare is both time point and time span

Discourse around jump scares often centers around the time point of the visual intrusion, the acoustic stinger, and the elicited startle response, suggesting that the jump scare has little to do with what occurs previously. This view suggests that jump scares differ only by the magnitude of the resulting SR. And yet, from a perceptual perspective, the SR produced by a jump scare can be inhibited, potentiated, or otherwise altered based on audience expectation. To be sure, the formation of expectancies leading up to and directly following the startle response call into question the presumed “rudimentary” reaction of the jump scare startle (Nummenmaa, 2024, p.13).

In light of recent theories of expectation, the magnitude of the SR in response to a jump scare may reflect sensory (or psychoacoustic) processing mechanisms that differ very little across audiences, as well as cognitive processing mechanisms associated with the statistical learning of commonly occurring events in the horror genre (Hutchings, 2009; Kirkham et al., 2002; Reber, 1989; Reber et al., 2019; Saffran et al., 1996, 1999). (Kurby and Zacks 2008) have argued, for example, that expectancy violations allow audiences to segment discrete “chunks” of relevant information (which they term *events*) from a continuous stream of stimuli (see also Baddeley, (1983). This concept of event segmentation has implications for perception, attention, and working memory (Jones et al., 1990; Jones and Morris, 1992).

Concepts related to the segmentation of temporal experience and the formation of schematic expectations have already been fruitful for investigating how audiences process films (Boltz, 2001; Cohen, 1999, 2013; Kurby and Zacks, 2022). Within the context of jump scares and horror films, how might frightening or otherwise surprising stimuli influence the formation of event boundaries or the retrieval of temporal event sequences within current theories of event segmentation (e.g., event horizon model; Radvansky, 2012)? The formation of expectations is also especially important for building tension. Previous studies have shown, for example, that the degree to which an audience anticipates an impending jump scare can increase the magnitude of the SR (Grillon et al., 1993), and the degree of reported fright (Cantor et al., 1984). Both findings indicate that the lead-up to a jump scare within the threat scene may be as important as the jump scare itself when predicting the magnitude of the subsequent response. From this point of view, does the formation of expectations preceding a jump scare depend on previous exposure to similar event structures stored in long-term memory? To be sure, this question continues to animate the experimental literature in music processing (Sears, 2025), but comparatively less is known about the sensory and cognitive mechanisms surrounding jump scares and the SRs they elicit.

**Figure 1:**
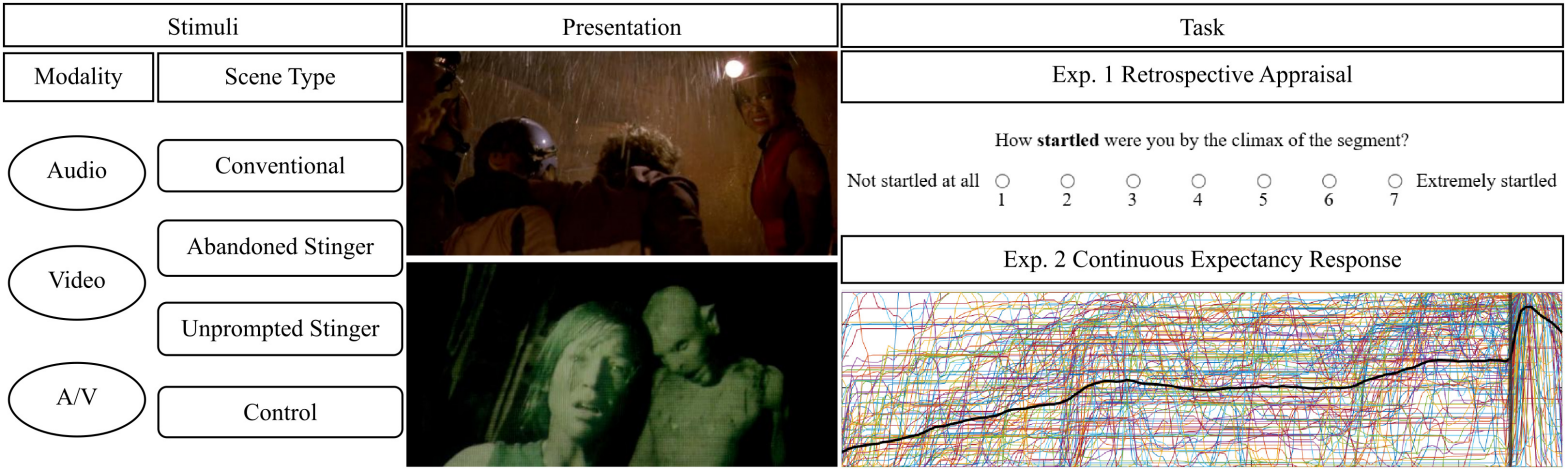
Two-experiment study examining cognitive mechanisms related to the processing of jump scares. Scene type conditions were determined based on four theorized temporal stages: the film's baseline audiovisual levels, the anticipation stage, the arrival stage characterized by the onset of the stinger, and the return to the baseline, after which the cycle may be repeated. The proposed conventional model contains all four stages. However, a jump scare scene may instead feature an abandoned stinger that prepares the audience for a jump scare via the baseline and anticipation stages but then omits the stinger and returns to the baseline stage, or an unprompted stinger that omits the anticipation stage and proceeds directly to the stinger, thereby denying the audience any preparation for the stinger. Film stills and continuous response data were taken from an unprompted stinger scene in The Descent (Marshall, 2005).

To address these issues, we conducted a series of experiments that examine the cognitive mechanisms related to the processing of jump scares (Acosta and Sears, 2025, under review). Summarized in Figure 1, a two-experiment study exposed participants to three categories of threat scenes as well as a control condition consisting of a non-threat scene from a horror film. Stimuli were also presented in three modality conditions (*audio only, video only, audiovideo*). Following previous studies, participants provided continuous ratings of expectation for a jump scare throughout the threat scenes (Sears et al., 2020; Steinbeis et al., 2006), as well as retrospective ratings of emotional appraisal related to their degree of startle, distress, tension, enjoyment, and expectation (Cantor et al., 1984).

Results suggested that particular audiovisual cues contribute to an *anticipation stage* within threat scenes that primes participants for an impending jump scare, regardless of whether the jump scare actually occurs. Conversely, jump scares that did not include anticipatory cues were reported to be less expected and more startling. Ultimately, our findings suggested that of the formation of multimodal expectations for jump scares in horror films may affect the resulting SR and its subsequent emotional appraisal. Future studies could adapt this paradigm to examine psychophysiological responses, incorporate individual differences in long-term exposure to horror, or further investigate whether these expectations ultimately contribute to the enjoyment of horror films.

## 5 A jump scare is multivalent

As mentioned previously, the literature typically associates jump scares with negatively valenced emotion terms like fear, fright, or terror (e.g., Sbravatti, 2019). Understandably, then, the experimental literature surrounding motivations for viewing the horror genre often asks, “why would sane people pay for a seat at a movie that they knew would scare them senseless?” (Tamborini and Stiff, 1987, p. 416).

Paradoxically, the moments directly following a jump scare can also elicit appetitive responses to aversive stimuli, thereby engendering both positively *and* negatively valenced emotions. Huron (2006) characterizes this experience as one of *contrastive valence*, wherein the brain rewards and reinforces correct predictions even for negatively valenced events. In this sense, while watching a horror film, the brain rewards correct predictions for the impending jump scare, just as it might for negatively valenced events in other domains like music (Koelsch et al., 2019).

This seemingly contradictory relationship becomes more intuitive given the potential motivations for selective exposure to horror (Martin, 2019; Scrivner et al., 2021; Sparks, 1986; Tamborini and Stiff, 1987). A positive reward is more appealing if the potential danger to the audience is entirely manufactured. Although audiences may respond to the film as though the danger is real (e.g., by experiencing increases in adrenaline and muscle tension following a startle response), they are nonetheless aware that the film is a constructed reality, which mitigates the threat of the potential consequences associated with incorrect predictions (Huron, 2006; Tan, 2008). This situation also distinguishes the jump scare from other invocations of the SR in nonaesthetic contexts, such as a backfiring car or a popping balloon, in which the threat of bodily harm is potentially real. Therefore, jump scares may be useful not only for studying SR responses to fear or disgust, but also for studying the enjoyment of, or selective exposure to, seemingly negatively valenced stimuli (e.g., horror films, violent video games, etc.).

## 6 Conclusion

In sum, the jump scare is a complex aesthetic experience that remains ripe for further research. As part of film, it remains richly multimodal and multidimensional, incorporating music, sound, and visual imagery. From an experimental perspective, the concept of the jump scare therefore offers researchers an ecologically valid paradigm to explore topics such as the startle response, the induction of emotions, and selective exposure to negatively valenced stimuli. How might the formation of expectations, for example, modulate the resulting SR? How might repeated voluntary exposure to horror films affect these responses? What is it about jumps scares that attracts audiences to (or at least does not dissuade them from) horror films? As Baird (2000) argues, there is indeed something “complicated and odd” underlying jump scares that deserves further study (p. 13). By outlining how jump scares are (1) an aesthetic experience that provokes a behavioral response, (2) multimodal, (3) both time point and time span, and (4) multivalent, we believe our current understanding of jump scares merely scratches the surface of what remains a persistently enigmatic and seemingly paradoxical aesthetic experience.
